# Report of the Experience of Living with High Blood Pressure in Light of the Theory of Caring

**DOI:** 10.17533/udea.iee.v38n2e10

**Published:** 2020-07-10

**Authors:** Juan Pablo Álvarez Najar, Mery Luz Valderrama Sanabria, Amalia Priscila Peña Pita

**Affiliations:** 1 Nurse, Masters. Professor, Universidad Pedagógica y Tecnológica de Colombia; Tunja, Colombia. Email: juanpablo.alvareznajar@gmail.com Universidad Pedagógica y Tecnológica de Colombia Universidad Pedagógica y Tecnológica de Colombia Tunja Colombia juanpablo.alvareznajar@gmail.com; 2 Nurse, Masters, PhD candidate. Associate Professor, Universidad de los Llanos; Villavicencio, Colombia. Email: mvalderrama @unillanos.edu.co Universidad de los Llanos Universidad de los Llanos Villavicencio Colombia mvalderrama @unillanos.edu.co; 3 Nurse, Masters. Professor, Universidad de los Llanos; Villavicencio, Colombia. Email: priscila.pena@unillanos.edu.co Universidad de los Llanos Universidad de los Llanos Villavicencio Colombia priscila.pena@unillanos.edu.co

**Keywords:** hypertension, self care, nursing theory, qualitative research., Hipertensión, Autocuidado, teoría de enfermería, investigación cualitativa., hipertensão, autocuidado, teoria de enfermagem, pesquisa qualitativa.

## Abstract

**Objective.:**

To analyze the report of the experience of the person with high blood pressure, in light of the theory proposed by Kristen Swanson,

**Methods.:**

This was a qualitative research with autobiographical-type narrative design. To collect and analyze the information, the work used open in-depth interview with 12 individuals, participant observation, and field notes.

**Results.:**

The study recognized the beliefs, customs, and cultural practices of the person living with high blood pressure and identified the care needs. The participants shared their feelings, finding that each confronts this disease differently and learns to care for themselves in particular manner**.**

**Conclusion.:**

In the analysis of the narratives of the experience of people with high blood pressure, five care processes by Swanson were recognized: maintaining the beliefs, knowing, being with, doing for, and permitting.

## Resumen

**Objetivo.:**

Analizar el relato de la experiencia de la persona con hipertensión arterial, a la luz de la teoría propuesta por Kristen Swanson,

**Métodos.:**

Se trata de una investigación cualitativa con diseño narrativo tipo autobiográfico. Para la recolección y análisis de la información, se utilizó la entrevista abierta a profundidad, a doce personas, la observación participante y las notas de campo.

**Resultados.:**

Se reconocieron las creencias, costumbres y prácticas culturales de la persona que vive con hipertensión arterial y se identificaron las necesidades de cuidado. Los participantes compartieron sus sentimientos, encontrando que cada uno afronta esta enfermedad de manera diferente y aprende a cuidarse de forma particular**.**

**Conclusión.:**

En el análisis de las narrativas de la experiencia de las personas con hipertensión arterial se reconocieron los cinco procesos de cuidado de Swanson: mantener las creencias, conocer, estar con, hacer por y permitir.

## Resumo

**Objetivo.:**

Analisar o relato da experiência da pessoa com hipertensão arterial, à luz da teoria proposta por Kristen Swanson,

**Métodos.:**

Se trata de uma investigação qualitativa com desenho narrativo tipo autobiográfico. Para a coleta e análise da informação, se utilizou a entrevista aberta a profundidade, a doze pessoas, a observação participante e as notas de campo.

**Resultados.:**

Se reconheceram as crenças, costumes e práticas culturais da pessoa que vive com hipertensão arterial e se identificaram as necessidades de cuidado. Os participantes compartiram seus sentimentos, encontrando que cada pessoa afronta esta doença de maneira diferente e aprende a cuidar-se de forma particular**.**

**Conclusão.:**

Na análise das narrativas da experiência das pessoas com hipertensão arterial se reconheceram os cinco processos de cuidado de Swanson: manter as crenças, conhecer, estar com fazer por e permitir.

## Introduction

Currently, high blood pressure (HBP) has become one of the first causes of morbidity and mortality globally. According to the WHO, in the world there are 1.130-billion people with this disease. By 2015, one in every four 4 men and one in every five women had hypertension; the purpose is to reduce HBP prevalence by 25% by 2025.(1) Due to the high number of patients and its complications that can cause death, the pathology is considered a public health problem throughout the world, becoming a risk factor for high-cost chronic and degenerative diseases, like cerebrovascular, cardiac, arteriosclerosis, and renal diseases, among others.(1) High blood pressure demands strict regular and constant compliance with the therapeutic regime, which implies changes in life habits because the treatment has a pharmacological component and a non-pharmacological one (diet, physical activity, stress management). However, most patients abandon the recommendations from the health staff as of the first year after being diagnosed because they believe they are feeling better, forget to take the tablets, or get tired with the treatment.(2) Also, health professionals and the very health system face the problematic of the lack of adherence to the treatment.(3)

Knowledge patients have acquired about the disease and treatment lets them act consciously,(4) bearing in mind the characteristics, effects, risks, and adequate behaviors to manage HBP. The selection of the diet(5-7) in elderly adults is of great importance for the patients’ care, given that it permits their obtaining a balance in their health status to avoid complications. Regarding the perception of life of the patient with HBP, it changes upon knowing of their diagnosis; at the start, the reaction tends to be of rejection and resistance to adopting other eating habits and lifestyle;(2) thereafter, they reflect and know they will have to care for themselves for the disease not to cause complications.(4) However, there are patients who do not modify living conditions.(6) From the emotional sphere, diverse feelings are experienced that often the health professional is not capable of understanding; each being is unique and must be cared for independently.(7) Beliefs with respect to managing the disease play an important role when establishing care guidelines, as well as the culture with the life habits.(8)

In nursing, theoretical referents exist that are part of the essential component of the discipline, one of them is the theory of caring by Kristen Swanson,(9) which represents care based on the maintaining belief in patients, anchored on knowing the reality of the other, transmitted through being with, and enacted by doing and enabling. 

It was deemed pertinent to use this theory in the research, given its relevance for the nursing discipline and thinking on the wellbeing of the individual with HBP to achieve manifesting their feelings, thoughts, and behaviors. Additionally, using this theory will permit moving toward taking care actions in this type of individuals, starting from the fact that they tend to have difficulties in finding the relationship between the theoretical referents and the practice. The study sought to analyze the report of the experience of the person with high blood pressure in light of the theory of caring. 

## Methods

This was a qualitative research with autobiographical narrative-type design,(10) applying the theory of caring. This project was carried out from June to August 2018 in the San Vicente de Ramiriquí Hospital in Boyacá, Colombia. The sequence of actions considered to approach the narrative study was thus: (i) *Election of the context and participants*: these were contacted with the database provided by the manager at the San Vicente de Ramiriquí Hospital, in the municipality of Ramiriquí in the department of Boyacá (Colombia); (ii) *Immersion to the field* was conducted through recognition and subsequent revision of the site to recreate the life history, experiences, and events; (iii) *The Selection of participants* agreed on attending the meeting of the Program of caring for chronic patients held once a month in the health institution. The research objective and the guarantee of confidentiality of the information shared were explained. The work also consulted the clinical histories to enrich the study; (iv) *Application of the theory* by Kristen Swanson, explored the meaning of the experiences narrated and documented. The first story was developed to collate and cross it with sources from the theory, verifying the facts and perspectives from the different participants; and (v) *Units, categories and themes of the narratives,* establishing coherence between that said in the interview and how it was said through observation and field notes.

The report was made, then these results were revised by experts in qualitative research and who have applied theories; thereafter, it was returned to the participants to introduce the definitive version. The sampling was selective, judgmental, or intentional because it offered in-depth and detailed information on the purpose of this research. Inclusion criteria considered elderly adult patients diagnosed with HBP for at least six months prior to conducting this study, who voluntarily participated in the research. Some were approached personally, after attending the scheduled medical control and others via telephone. Initially, fifteen patients were summoned, two of which did not accept to participate due to work occupations and one withdrew during the middle of the study due to personal reasons. With this group, the first interview exercise was begun. The narratives were transcribed exactly as the person expressed them, in maximum spontaneity, giving as much freedom as possible. To preserve the identities of the participants and give the reports poetic nature, their names were changed for those of some flowers.

To gather and analyze the information, which were conducted simultaneously, the open, in-depth interview, participant observation, and field notes were used. The hospital director provided a room for the meetings with the participants; it was a place with privacy and tranquility for the participants. During the interview, the researchers supported on their experience, carried out several extensive sessions (on average, three meetings with each person) and detailed, starting with guiding questions, like: How do you care for yourself since you were diagnosed with HBP?, Do you know what is the cause of your disease? According to the answer, new questions were made. These were recorded and transcribed textually with prior signing of the informed consent, agreeing on the meeting with the participants. Three meetings took place, in each of them the story was again addressed and progress was made in the depth of the story. The interview lasted from 30 to 60 minutes. A conscious position was assumed, placing trust on the person telling the story.

The techniques and analytic procedures mentioned permitted the researchers to develop a substantive theory, compatible with the phenomenon observed. The analysis was a cyclical, flexible, dynamic and creative process, while systematic. The concepts were denominated and categories defined, through the constant comparative method. Coding was performed at three levels:

(i) *Open coding*: this level performed an initial descriptive analysis of the codes (specific words of the participants) and of line by line and phrase by phrase, looking for words to generate codes and define categories. The data were compared and each interview with the previous, underscoring relevant words and phrases that expressed aspects of the phenomenon under study; (ii) *Axial coding:* this level identified, the relationships between the codes and categories. Hence, the data was again joined through induction and abstraction to establish connections between codes and categories, similar categories were grouped and new data with emerging codes to construct initial relationships; and (iii) *Selective coding:* this level integrated the categories, in search of a central one, to construct a larger theoretical scheme. Triangulation of information was carried out when calling on other informants, databases from the health institution, and personal documents, like clinical history, in search of the confirmation.

To grant validity to the research, rigorous methodological criteria were kept in mind during the research, such as credibility, dependency or consistency, and confirmability. The research was approved by the ethics committee at the San Vicente Hospital in Ramiriquí, dated 20 June 2018. Ethical principles were kept in mind with the signing of the informed consent. 

## Results

This study had the participation of 12 patients (7 men and 5 women), ages ranged between 50 and 91 years. Nine of them have endured the disease for over five years; 40% live with their children; 35% live with their spouse and children; and 25% with other relatives. The following will describe the principal research results, according to the five caring processes proposed by Swanson.(9)

### Maintaining Belief

The orientation of caring for patients with HBP stems from a fundamental belief in them and their capacity to overcome obstacles imposed by the disease. Elderly adults tend to have physical and emotional changes in their health status; the fact of living with HBP faces them with challenges they must confront to learn to assimilate the disease. Among the caring beliefs, they recognize those of religious type and others that have been passed on from generation to generation from home practices: *I have learned to prepare my remedies, take them, then I go to the medical check-up and keep the tension controlled, mandarin juice and that of apple with spinach and celery relieve my stress and lower my tension* (Alhelí, female, 56 years)*; Well I say… what can we do now! Those are the evils of old age and you must adapt to everything; I have accepted positively this disease, these are the things God sent us, I faced reality and learned the most-serious consequences to raise awareness* (Narciso, male, 77 years).

*I tell you, sometimes I drink the herbal teas with lemon, with lots of faith for them to do me good, let God’s will be, whatever He wants for me. I use pork lard and rub it where it hurts. For example, if my heart aches, I rub it there, but I have to do it immediately once I get the discomfort because if I do it later it does not work; I apply a little on my forehead in two crosses with massage and it goes away.* (Orquídea, woman 91 years); *the truth is that I use home remedies, figs, parsley leaves and lemon balm, all that to make herbal tea. I also use lemon bee brush when my nerves are altered. I confess that the disease has affected me tremendously, given that I cannot do the same as before, every year I use to raise four cattle in the moor farm, and now I only have one cow and a calf in the paddock, I can no longer take care of them the same way, I even had to sell the farm to burry my husband and a son and some money was left over and I have it in the bank for the children to study* (Violeta, female, 73 years); *I use garlic, I peel them, chop them up and eat them raw with water, I use two or three garlic cloves each time my pressure rises; when I eat them, I feel I improve and the fast beating of my heart stops.* (Azucena, female, 69 years).

### Knowing

Understanding the patients’ lives permits recognizing knowing their care needs, which have to do with the lack of information about the disease and its treatment, as well as the importance of comprehending and respecting the eating habits and benefits generated by complying with the medical prescription and recommendations offered by the health staff. This is a compromise between the care giver and the subject of care: *For me, going to the hospital is a routine procedure, they care for me, give me a prescription, I claim the medications and leave to continue with my tasks. I prefer not to ask so that I am not kept late, that is why I really do not know what is the cause of this disease and why they give me so many medications* (Clavel, male, 51 years)*; They always make me arrive 10 minutes before the medical consultation, I need to rest for five minutes, then they take my vital signs and weigh me. After this, they send me with the massage doctor and with the nurse to perform some exercises* (Gardenia, female, 68 years)*; When I go to the medical consultation, they ask me how I’m feeling; if I tell them I am well, they prescribe medications for three months, but only give me enough for one month. Then I go claim my medications, there they give me some recommendations. I always look at the color, size, and shape of the pills, but do not stop to look at the name. They explain which ones I have to take in the morning and which ones in the afternoon (Azucena, female, 69 years); Sometimes I am not satisfied with the care because I’ve told the doctor that the yellow pill does nothing to me, but he keeps prescribing it, I only take the red ones when I start to get a headache, but I don’t have enough information of what happens to me* (Geranio, male, 68 years); *learning to eat without salt has been somewhat difficult; however, I have gotten used, I try to eat fruits, vegetables and sometimes I take the skin off the chicken, but I won’t refuse to eat a good farm chicken. I’m told I have to exercise, but the truth at this age I don’t like to, sometimes I get scared because my tension rises sky high* (Tulipán, male, 79 years); *I want to tell you that I do my massages, if my tension rises I make two crosses on the forehead and lie down a while and when I feel normal I get up again to do my chores* (Orquídea, female, 91 years)*.*

### Being with

The participants managed to share some feelings and trust was generated with the researchers who understood their specific situation. Certain fears were highlighted, especially that of death as something unexpected for some and predictable for others. The relationship established permitted motivation to know more about the disease: *you have to die of something, I can’t tell you what because, for example, doctors have told me I’m dying of hypertension or through a heart attack or of a stroke, of those three things I might die. Every day when I lay down, I think about death, we don’t know the day or moment, but it will arrive.* (Orquídea, female, 91 years); *I almost can’t go anywhere alone, I am afraid; I have fainted at home, I already paid for a mass to ask God to help me, He is the biggest doctor.* (Violeta, woman 73 years); *I do not fear death, if it arrives, it arrives, someday it must be, you don’t have to fear it. It is like a plant, it is born, grows, reproduces, and dies and that’s how we are, sooner or later the moment arrives. I see many who want to die, but death arrives when Dios wants not when you want* (Crisantemo, male, 63 years).

### Doing for

Each individual copes with the disease differently: some seek support from the family and consolation from friends, while others are more reserved with the health situation: *I usually don’t tell my family what happens to me, I only get up and take my medications or me rub myself because I fear causing them worry and stealing their peace at work and study, I prefer to stay quiet and place myself in the hands of God, which cures all evil, sometimes a neighbor comes to visit and she listens to my regrets* (Orquídea, female, 91 years)*; For me, it has been very hard to live with this disease since I was diagnosed, but over time I began to assimilate it. My children have become great support and, thus, everything has been a little more bearable.* (Gardenia, female, 68 years); *I have gotten used to the hypertension, with God’s help and especially my family that always supports me to deal with this disease. As I said, in this you must have will power, although sometimes you want to give up, but, finally, everything is a habit, but I get tired of taking so many pills (medication)* (Clavel, male, 51 years).

### Enabling

This process was evaluated from two aspects; on the one hand, self-care actions were discovered and on the other hand, it favored the expression of opinions regarding the disease: *I know I should take the medications for life and at the time indicated by the physician; on the contrary, this disease will worsen and I can die. I also stopped eating so much salt, I am mindful of what I eat, now I am more aware that I must do my part, I am checked by some doctors and the nutritionist. They also told me to exercise, but sometimes my legs hurt too much* (Lirio, male, 73 years); *Now I behave more, I do exercise and walk every day, I’ve lost weight and that has also helped me to control the disease, my daughter tells me I should do what the doctor says, go to controls and watch my eating. However, I don’t like eating without salt, I cut back on carbohydrates, eat vegetables, and drink lots of liquids especially water (*Geranio, male, 68 years).

*Since I got the disease, I never touch the salt shaker, not even to eat an egg, but I don’t have time to do exercise. I try to take the pills at the times ordered, I keep them in my shirt pocket, but sometimes I forget, then I take them until I remember* (Narciso, male, 77 years); *I stopped drinking alcohol because I used to drink with my husband, two shots in the morning, after lunch and at night given that we had a grocery store we also drank with friends, but not to get drunk only moderately. Alcohol is poison; I also consumed too much hot sauce. Once I ate fried foods with lots of hot sauce and suddenly a vessel exploded (a blood vessel burst). I was taken to the hospital and the bleeding would not stop. (Violeta, female, 73 years); No more salty potatoes, juices blended in sweets because my sugar level will rise and the cholesterol and alcohol has been difficult to leave; it was always hard to change my feeding habits. I also had to change friends and bad company doesn’t bring anything good* (Violeta, female, 73 years)*; I think I got this disease by eating too much salt and eating too much fat, I loved fried foods, junk food and cooked with lots of salt, I also ate fried pork, greasy soups and many carbohydrates, besides, at home we were always fighting* (Jacinto, male, 75 years); *I got this disease due to the loss of my son, sadness and desperation overtook me and since then I got sick, stress at work was terrible and that also raised my tension* (Gardenia, female, 68 years); *I think I got sick because I was not exercising or walking, given that I spend my time sitting, I do not engage in any activity to improve my pressure* (Tulipán, male, 79 years). 

## Discussion

Through Swanson’s theory, the human is perceived as a unique person, manifested through feelings, thoughts, behaviors, and life experiences.(9) According with the testimonies presented in each process, it should be highlighted that *Maintaining beliefs* occurs through religious practices and use of traditional medicine with home treatments to control blood pressure.(11) The population in Boyacá has strong cultural roots that could cause lack of adherence to the pharmacological treatment. This concept coincides with the results in the study by García, R *et al*., which found big benefits in using natural remedies compared with the pharmacological treatment, in addition to being safer because they do no harm in the organism.(12) Another recommended use also accepted by the participants in the research is garlic to diminish blood pressure.(13,14) However, the effect changes according to how it is taken, the best way is in extract because it increases the concentration of S-allyl-cysteine that is tolerable and functional; rather, when eating it whole, one of its components denominated Allicin, is volatile and unavailable.(14) According to the aforementioned, it is possible to state that people’s beliefs along with the biological, cultural, and family factors are important to assimilate the treatment, hence, the importance of establishing agreements between the patient and the health staff about the treatment’s parameters.(15) 

Regarding *Knowing*, patients state that they have little information about the disease and the reason for the treatment, but try to comply with it; this coincides with García *et al*., and Ried, F, who conclude that users try to obey the medical prescription even if they do not strictly follow the indications,(12,16) or as stated by a participant, they take the medications only in case they have any symptom (dizziness or headache) or when they have high blood pressure.(17) Learning to eat without salt has been a difficult habit to acquire, is one of the testimonies in this study that coincides with Negreiros and Asakura who report the low-sodium diet as one of the practices with greatest inconvenience to follow the treatment.(18) Literature reports the benefits related to physical activity;(19) however, in this study it was not found practiced by all the participants. 

Listening and sharing the individual’s feelings of concern and sadness are included in *Being for,* which reported fear of death and the worry caused by having a serious disease. The affliction, suffering and fear of having BPH are also expressed as a threat to life that leads to suffering with resignation.(5,17)

*Doing for* was manifested through consolation and support from the family, neighbors, and friends. This coincides with Moura *et al.,* regarding support from relatives and friends complying a role in adapting to new habits in hypertensive patients, thereby, strong commitment is required from everyone.(20^,^^21^)

The analysis of the concept of *Enabling* described the self-care actions; one of them is to walk every day. This practice is considered as low-cost and minimum-risk physical activity that most humans can perform. Evidence shows that it offers positive effects in lowering systolic pressure, diastolic pressure or both.(22) The participants expressed their opinions to protect health, with diminished intake of salt and fats and moderate practice of physical activity. It is necessary to participate self-care to comply with the treatment,(23) some of the practices include diminished salt intake, increased intake of fruits and vegetables, and enhanced physical activity.(24-26) Many patients have modified their eating habits by following the recommendations by the health staff, in spite of not agreeing and that it is not pleasant to their taste; the changes consist principally in diminishing intake of fried foods, sugar, and salt and adding fruits; the whole family joins these new habits.(7)

This study concludes that in the analysis of narratives of the experience of patients with high blood pressure, the five caring processes by Swanson were recognized. The integral assessment nursing makes of these individuals is fundamental, respecting their beliefs, learning to know it, sharing their feelings and fears as fundamental concepts of care and, thus, permitting to advance in the disciplinary development. 
